# The involvement of follistatin-like protein 1 in osteoarthritis by elevating NF-κB-mediated inflammatory cytokines and enhancing fibroblast like synoviocyte proliferation

**DOI:** 10.1186/s13075-015-0605-6

**Published:** 2015-04-02

**Authors:** Su Ni, Kaisong Miao, Xianju Zhou, Nanwei Xu, Chenkai Li, Ruixia Zhu, Rongbin Sun, Yuji Wang

**Affiliations:** Department of Orthopedics, The Affiliated Hospital of Nanjing Medical University, Changzhou No.2 People’s Hospital, 29 Xinglong Alley, Changzhou, 213003 China; Laboratory of Clinical Orthopedics, The Affiliated Hospital of Nanjing Medical University, Changzhou No.2 People’s Hospital, 29 Xinglong Alley, Changzhou, 213003 China; Department of Neurology, Laboratory of Neurological Diseases, The Affiliated Hospital of Nanjing Medical University, Changzhou No.2 People’s Hospital, 29 Xinglong Alley, Changzhou, 213003 China

## Abstract

**Introduction:**

Our previous work has revealed that expression of follistatin-like protein 1 (FSTL1) is elevated in the synovial tissues from osteoarthritis (OA) patients. The aim of this study was to elucidate the underlying molecular mechanisms by which FSTL1 plays a role in the pathogenesis of OA.

**Methods:**

Cultured fibroblast-like synoviocytes (FLSs) from synovial tissues of OA patients were stimulated with human recombinant FSTL1, and then the expression of inflammatory cytokines in FLS and their concentrations in the cell supernatants were measured by real-time polymerase chain reaction (PCR) and enzyme-linked immunosorbent assay (ELISA), respectively. Nuclear factor kappa B (NF-κB) activation was examined by western blot and chromatin immunoprecipitation (ChIP) assay at the p65 binding site. Finally, the proliferation of FLSs and the expression level of the proliferation-related tumor suppressors (p53 and p21) were determined by MTS assay kit and western blot in the presence or absence of FSTL1, respectively.

**Results:**

FSTL1 remarkably promoted expression levels of several inflammatory cytokines (tumor necrosis factor alpha (TNF-α), interleukin-1β (IL-1β) and interleukin-6 (IL-6)) *in vitro*. Western blot analysis showed that FSTL1 activated the inflammatory-related NF-κB signaling pathway, as validated by ChIP assay detecting p65-binding level on the cytokine promoter region. Moreover, FSTL1 promoted the proliferation of OA FLS by downregulating the expression of p53 and p21. Interestingly, the concentration of synovial fluid IL-6 was remarkably elevated in OA patients, and was correlated with synovial fluid and serum FSTL1 levels.

**Conclusions:**

These findings show that FSTL1 functions as an important proinflammatory factor in the pathogenesis of OA by activating the canonical NF-κB pathway and enhancing synoviocytes proliferation, suggesting that FSTL1 may be a promising target for the treatment of OA.

## Introduction

Osteoarthritis (OA) is a rheumatic disorder with the highest prevalence among all arthritis. Knee OA causes to most of mobility disability in the elderly over 65 [1]. As the degradation of articular cartilage proceeds, OA patients suffer from a variety of symptoms including joint pain, tenderness, stiffness, locking, and sometimes an effusion. Importantly, the synovium and cartilage of the OA patients are often subject to extreme changes of microenvironment triggered largely by inflammatory cytokines and proteases in extracellular matrix (ECM), and thus leading to dysfunction of various types of cell in synovitis joints, especially fibroblast-like synoviocytes (FLSs) [2,3]. Inflammation of the synovium is frequently involved in the pathological process, even in the early stages of OA [4,5]. A growing body of evidence indicates the importance of activated FLS in OA pathogenesis. FLS promotes cartilage degradation through the secretion of inflammatory/catabolic mediators and other soluble factors [6-8]. Therefore, cultured FLS is a good *in vitro* model for OA pathogenesis and drug target discovery [6,9].

Among multiple pathways and mediators influencing the development and persistence of OA, nuclear factor-kappa B (NF-κB) transcription factor plays a prominent role [10,11]. The NF-κB family consists of P50 (NF-κB1), P52 (NF-κB2), P65 (RelA), RelB and C-Rel. NF-κB protein, a heterodimer of p50 and p65/RelA, is usually sequestered by inhibitor of kappa B alpha (IκBα) in the cytosol in the inactive state [12,13], NF-κB can be activated by proinflammatory cytokines, excessive mechanical stress and ECM degradation enzymes. Once the NF-κB signaling cascade is activated, a complete degradation of IκB following phosphorylation by upstream IκB kinases (IKKs) allows the translocation of the Rel/P50 homodimer to the nucleus and subsequently induces gene transcription [14,15]. Activated NF-κB boosts the expression of many inflammation-related cytokines and chemokines, such as interleukin 6 (IL-6), interleukin 8 (IL-8), adhesion molecules and matrix-degrading enzymes, and thus regulating inflammation, immune functions and cellular growth [16,17]. Therefore, NF-κB-activating kinases are one of the potential therapeutic OA targets [10].

Follistatin-like protein 1 (FSTL1), which was first identified as a transforming growth factor β1 (TGF-β1)-inducible protein, is a secreted extracellular glycoprotein containing a follistatin-like and extracellular calcium-binding domain [18]. It has been reported that FSTL1 is involved in diverse biological processes including cell proliferation and differentiation, wound healing, inflammation, skeletal muscle growth and fibrosis [19-21]. Previous studies revealed a substantial connection between the FSTL1 level and severity of rheumatoid arthritis [22,23]. Moreover, our past work showed that FSTL1 acts as a serum biochemical marker of OA and reflects the severity of joint damage in patients [24].

However, the underlying mechanisms by which FSTL1 plays a role in the pathogenesis of OA are poorly understood. In view of the crucial role of FLS in OA progression [2,11], this study was aimed at investigating the signal transduction of FSTL1 in cultured OA FLS, and revealing the potential roles of FSTL1 in OA pathogenesis.

## Material and methods

### Synovial fluid and serum samples

Serum and synovial fluid (SF) samples were obtained from 58 OA patients by synovectomy or joint replacement surgery. OA serum and SF samples were obtained from patients diagnosed with knee OA (of Kellgren-Lawrence (KL) score 1 to 4 [25]) according to the 1985 criteria of the American Rheumatism Association [26]. The clinical and demographic characteristics of OA patients and their controls are shown in Table 1. The SF samples obtained were centrifuged for 5 min at 10,000 rpm to remove the obvious contamination with blood or debris. A total of 30 serum samples of apparently healthy individuals diagnosed without alcohol abuse or chronic drug or other chronic disease or acute illness were included as healthy controls (HCs).This study was reviewed and approved by the ethics committee of Changzhou No.2 People’s Hospital. Informed consent was received from all patients and controls.

**Table 1:** **Characteristics of the subjects investigated** ^**a**^

	**HC**	**OA**
**Number**	30	58
**Age (years)** ^**b**^	60 (55–64)	66 (60–75)
**Sex (M/F)**	16/14	25/33
**Disease duration (years)** ^**b**^	-	1.0 (0.5–3.0)
**KL grade (0–4)**	-	0/11/14/23/10

^a^Included all subjects for whom serum samples were available, and all the SF samples and primary cell lines from OA subjects were available; ^b^expressed as the median (25th to 75th percentile). HC, healthy control individual; OA, osteoarthritis; KL, Kellgren-Lawrence; SF, synovial fluid.

### Isolation and culture of human FLSs

Synovial tissue specimens were obtained from OA patients by synovectomy or joint replacement surgery. Tissues were carefully minced and digested with 1 mg/ml collagenase I (Sigma-Aldrich, St. Louis, MO, USA) in serum-free Dulbecco’s modified Eagle’s medium (DMEM) (Gibco BRL, Grand Island, NY, USA) for 4 to 6 hrs at 37°C, filtered through a 70-μm cell strainer (BD, Durham, NC, USA), extensively washed with DMEM blank and finally cultured in DMEM supplemented with 10% fetal calf serum (Gibco BRL, Grand Island, NY, USA), 100 U penicillin, and 100 μg/ml streptomycin in a standard cell culture chamber containing 5% CO_2_. The nonadherent cells were removed next day. The adherent cells were cultured up to 90% confluence and then split in 1/3 ratio up to passage 3–6 (consisting of a homogeneous population). The cultured FLSs at these passages were used for subsequent experiments.

### Reagents and stimulation assays

Each FLS line from an individual OA patient was used for each experiment, which was repeated two times with different OA FLS lines. Cultured FLSs were grown in 100-mm cell culture dishes (8 to 10 × 10^5^ cells/dish) for western blot or in 6-well plates (1 to 1.5 × 10^5^ cells/well) for mRNA extraction. Cultures were stimulated with human recombinant FSTL1 (Adipo Bioscience, Santa Clara, CA, USA), which was dissolved in phosphate-buffered saline (PBS) solution, or vehicle (PBS solution). Stimulation was applied for 24 or 48 hrs for mRNA extraction, or for 30 min for NF-κB activation, or 48 hrs for supernatant collection. BAY 11–7082 (Selleckchem, Houston, TX, USA), a specific inhibitor of NF-κB signaling pathway, was dissolved in dimethyl sulphoxide (DMSO) solution to the concentration of 5 mM, and added to the cultures at ratio of 1:1,000 (final concentration was 5 μM) [27] for 1 hr pretreatment.

### Cell proliferation

Cell proliferation assay was performed using a novel tetrazolium compound (3-(4,5-dimethylthiazol-2-yl)-5-(3-carboxymethoxyphenyl)-2-(4-sulfophenyl)-2H-tetrazolium, inner salt; MTS) and an electron coupling reagent (phenazine ethosulfate; PES)-based CellTiter 96™ AQueous One Solution Cell Proliferation (MTS) assay (Promega, Madison, WI, USA). OA FLSs were seeded at about 2,000 cells per well in 96-well plates in triplicate for 7 days under regular growth conditions (DMEM with 10% fetal bovine serum (FBS)), and then MTS cell viability assay was performed daily according to the manufacturer’s instructions. Twelve hours after seeding, FSTL1 was added daily in the medium, which was changed every two days.

### Real-time polymerase chain reaction (PCR)

Total RNA was extracted from cultured FLSs using NucleoSpin RNA Kit (MN, Düren, Germany) according to the manufacturer’s instructions. One μg total RNA was reverse-transcribed using the High-Capacity cDNA Reverse Transcription Kit (Applied Biosystems, Foster City, CA, USA) according to the manufacturer’s instructions. A quantitative real-time PCR assay was carried out using SYBR™ Select Master Mix (Applied Biosystems, Austin, TX, USA) in a Bio-Rad iQ5 (Bio-Rad Laboratories, Hercules, CA, USA). Primer sequences for amplifying human cytokines cDNA and internal control glyceraldehyde 3-phosphate dehydrogenase (GAPDH) were as follows: IL-1β, 5′- AAGCTGAGGAAGATGCTG −3′ (forward) and 5′- ATCTACACTCTCCAGCTG −3′ (reverse); IL-6, 5′- GAACTCCTTCTCCACAAGCGCCTT −3′ (forward) and 5′- GACCAGTGATGATTTTCACCAGG −3′ (reverse); TNF-a, 5′- CCAGGCAGTCAGATCATCTTCTC −3′ (forward) and 5′- AGCTGGTTATCTCTCAGCTCCAC −3′ (reverse); GAPDH, 5′-AGGGCTGCTTTTAACTCTGGT-3′ (forward) and 5′-CCCCACTTGATTTTGGAGGGA-3′ (reverse). The comparative threshold cycle method was used for relative quantification of mRNA.

### Western blot

Cultured FLSs were lysed in RIPA and boiled. Sodium dodecyl sulfate (SDS)-polyacrylamide gel electrophoresis was conducted on 10% polyacrylamide gel and transferred to polyvinylidene fluoride (PVDF) membrane. Rabbit anti-human p65 polyclonal antibody, rabbit anti-human phosphate p-65 polyclonal antibody, rabbit anti-human IκB polyclonal antibody, mouse anti-human phosphate IκB monoclonal antibody, and rabbit anti-human p50 polyclonal antibody (all these antibodies were purchased from Cell Signaling Technology, Danvers, MA, USA) were used to detect the corresponding protein in the NF-κB signaling pathway. Rabbit anti-human actin polyclonal antibody was used to detect actin signal as an internal loading control and relative expression levels were quantified by running the Quantity One software (Bio-Rad Laboratories, Hercules, CA, USA).

### Chromatin immunoprecipitation (ChIP)

Cultured FLSs were subjected to 5 μg/ml FSTL1 or vehicle (PBS solution) for 12 hrs. A total of 10^7^ cells were fixed and cross-linked in fresh 1% formaldehyde for 10 min and then quenched with 2.5 M glycine for 5 min at room temperature. Cells were then harvested and suspended in lysis buffer. Simple ChIP™ Enzymatic Chromatin IP Kit (Cell Signaling Technology, Danvers, MA, USA) was used according to the manufacturer’s instructions. Chromatin was digested with micrococcal nuclease, sheared by sonication and then lysates were clarified by centrifugation at 10,000 rpm for 10 min at 4°C. The supernatant was incubated with ChIP-grade rabbit anti-human p65 polyclonal antibody (Cell Signaling Technology, Danvers, MA, USA) or normal immunoglobulin G (IgG) overnight at 4°C with rotation. After being pulled down with protein G agarose beads, the target protein-DNA complexes were sequentially washed several times and reverse cross-linked to elute DNA for the subsequent experiment. DNA was purified and real-time PCR was performed to evaluate the ChIP-enriched DNA. Primer sequences for detecting p65-binding tumor necrosis factor alpha (TNF-α) promoter region were 5′- ATATGGCCACACACTGGGGC −3′ (forward) and 5′- GGGCTTGGTGGCAGGCTTGA −3′ (reverse). IL-6 promoter region were 5′- ACCCTCACCCTCCAACAAAG-3′ (forward) and 5′- GCCTCAGACATCTCCAGTCC −3′ (reverse).

### Enzyme-linked immunosorbent assay (ELISA)

TNF-α, interleukin 1β (IL-1β), and IL-6 levels in cell supernatants were measured using a standard quantitative sandwich ELISA (eBioscience, Vienna, Austria). All analyses and calibrations were performed in duplicate. Optical densities were determined using an absorbance microplate reader (Elx808™ Bio-Tek Instruments, Winooski, VT, USA) at 450 nm. Gen5 Data Analysis software (Bio-Tek Instruments, Winooski, VT, USA) was used to analyze all data and depict the standard curve.

### Statistical analysis

Statistical analysis was performed using Prism (GraphPad Software, San Diego, CA, USA) and SPSS 17.0 software (SPSS Inc., Chicago, IL, USA). χ^2^ test was used for sex difference between OA patients and the controls; Spearman’s rank correlation test was used to evaluate the associations between serum/SF FSTL1 level and SF IL-6 level; the significance of other differences was evaluated using Student’s *t* test. *P* values less than 0.05 were considered significant.

## Results

### FSTL1 induces expression and secretion of inflammatory cytokines from OA FLS

**Figure 1:**
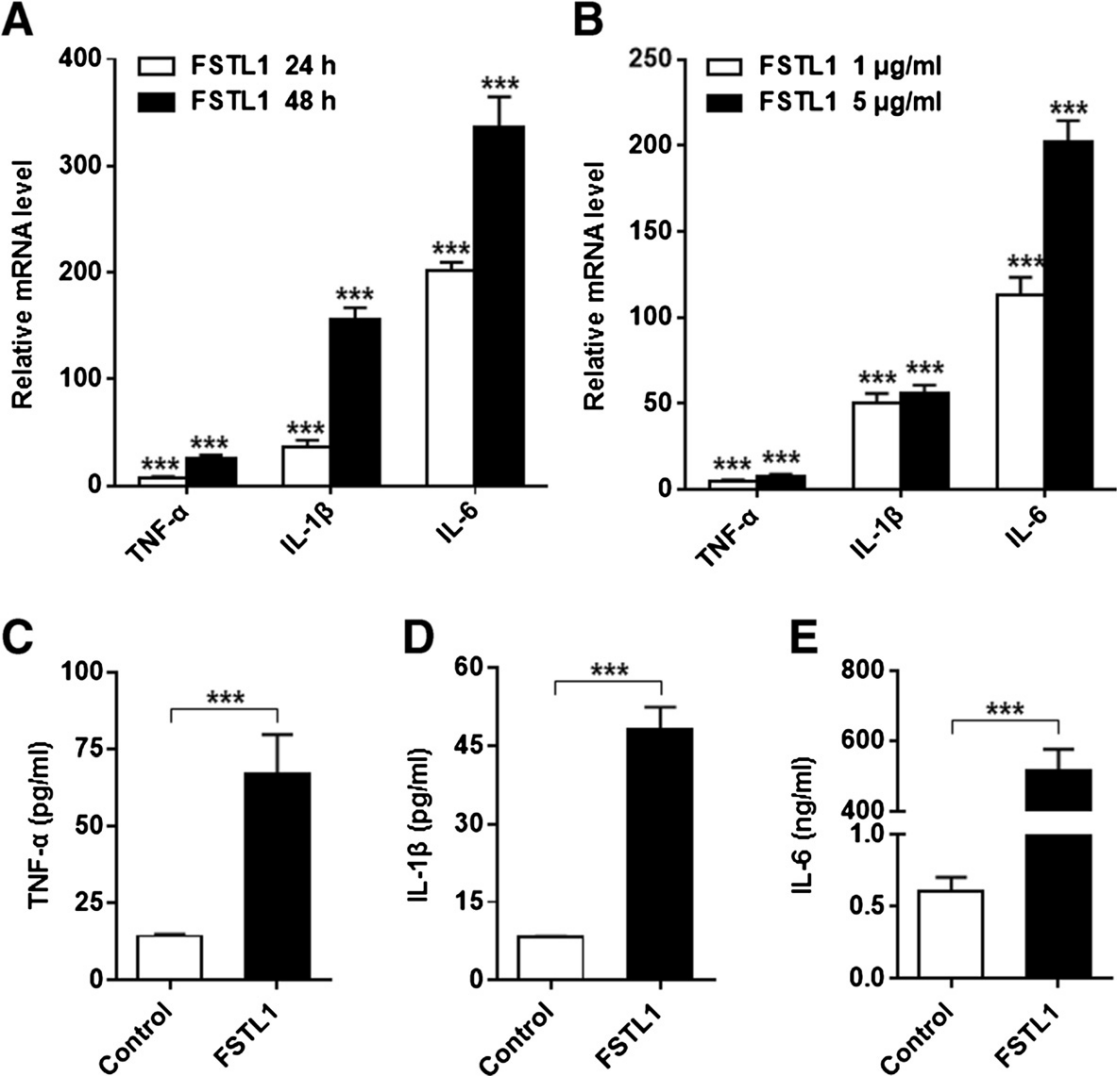
FSTL1 increases expression and excretion of inflammatory cytokines in cultured OA FLS. (**A**) Cultured FLSs were treated with FSTL1 (dissolved in PBS solution) in different concentration (1 μg/ml or 5 μg/ml) for 24 hrs. (**B**) Cultured FLSs were stimulated with 5 μg/ml FSTL1 for two different durations (24 or 48 hrs). In (**A**) and (**B**), total RNA was extracted and reverse transcribed, and the relative levels of TNF-α, IL-1β and IL-6 mRNA were analyzed by real-time PCR. The relative level of mRNA expression was normalized to the level of housekeeping gene *GAPDH* expression, and presented as fold changes relative to the control (the level of the control was set as 1). (**C**-**E**) Cell supernatant was collected from the cultures treated with 5 μg/ml FSTL1 for 48 hrs. TNF-α, IL-1β and IL-6 concentrations were measured by ELISA. The above results are presented as mean ± (standard deviation) SD of three independent experiments (each FLS line from an individual OA patient was used for each experiment). ^*^ *P* <0.05, ^**^ *P* <0.01, ^***^ *P* <0.001, compared to the control (the vehicle-treated group), Student *t* test. ELISA, enzyme-linked immunosorbent assay; FLS, fibroblast-like synoviocyte; FSTL1, follistatin-like protein 1; IL-1β, interleukin-1β; IL-6, interleukin-6; OA, osteoarthritis; PBS, phosphate-buffered saline; TNF-α, tumor necrosis factor alpha.

Previous studies showed that FSTL1 expression correlates with severity of arthritis both in OA patients and mice models [22,24]. And a connection between cytokine/chemokine level and FSTL1 expression has been established in mice arthritis models [22]. To further examine FSTL1 functions, we used primary FLSs from OA patients’ synovial tissues as model cells. First, we incubated FLSs with the two different FSTL1 concentrations (1 μg/ml and 5 μg/ml) for 24 hrs, we observed that FSTL1 remarkably increased the level of TNF-α , IL-1β and IL-6 mRNA in the FLSs in a dose-dependent way (Figure 1A). Surprisingly, the increment of IL-6 mRNA was extremely prominent (more than 200-fold). Moreover, 10 μg/ml FSTL1 treatment did not result in a further increase in these mRNA levels compared to the treatment with 5 μg/ml FSTL1 (data not shown). Next, FLSs were incubated with 5 μg/ml FSTL1 for two different times (24 and 48 hrs).The expression level of the three inflammatory cytokines increased with the incubation time (Figure 1B). Consistently, the three secreted protein levels in the supernatant were markedly elevated following 48 hrs of treatment with FSTL1 at 5 μg/ml (Figure 1C-E). Especially, elevation of released IL-6 was more than 500-fold. Together, these data further support our previous work that FSTL1 reflects the severity of OA by promoting the release of inflammatory cytokines in articular cavity

### FSTL1 activates NF-κB signaling in OA FLS

**Figure 2:**
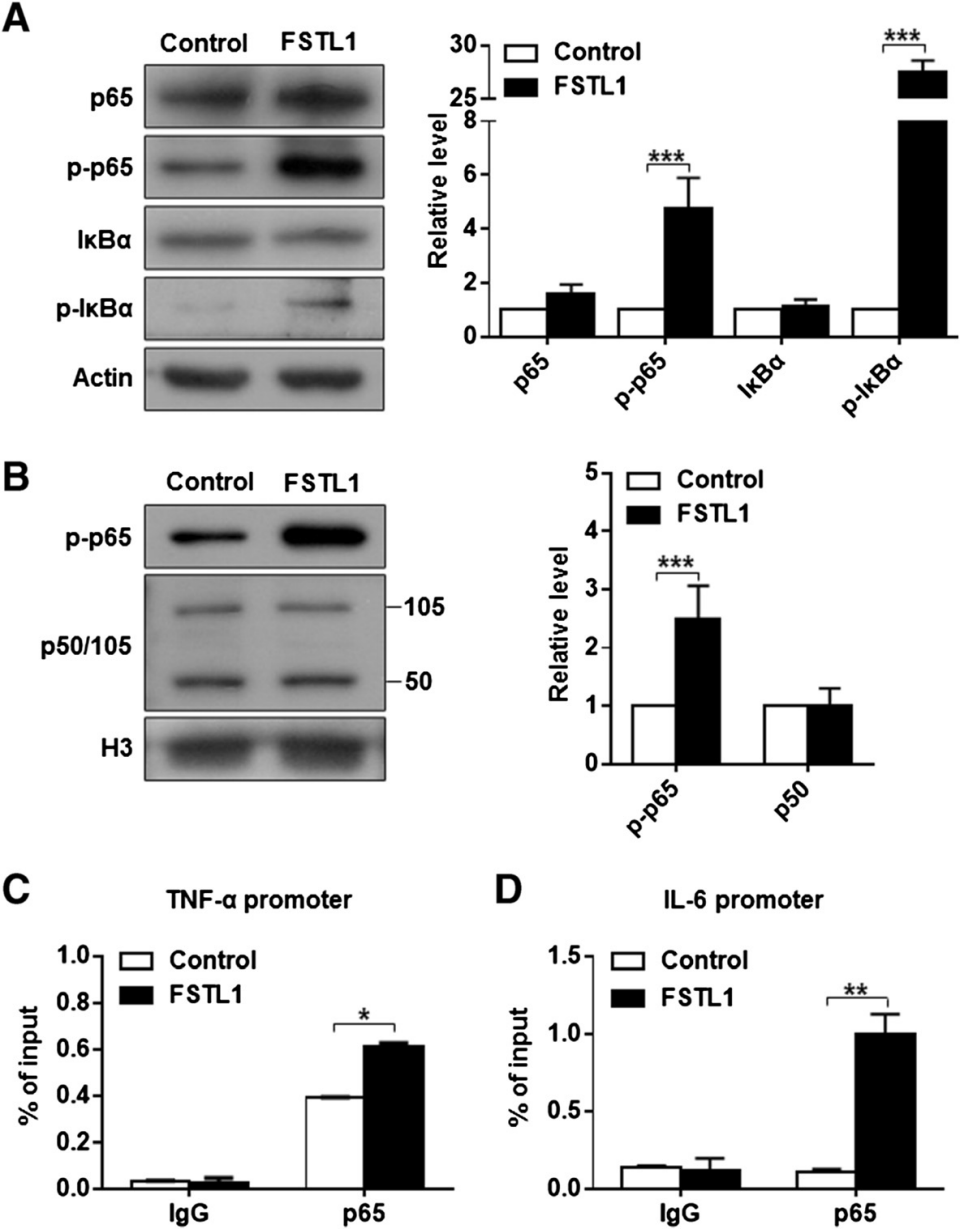
FSTL1 activates the NF-κB pathway in FLS. (**A**) Following treatment, the levels of key components of NF-κB in total cell lysates were determined by western blot analysis. (**B**) Nuclear extracts were isolated to further confirm the activation of NF-κB pathway. In (**A**) and (**B**), FLSs were treated with FSTL1 (5 μg/ml) or vehicle (PBS solution) for 30 min. Representative western blot (Left) and quantification data (Right) are shown for the corresponding groups. The relative protein levels were normalized to the level of the internal control, actin or histone 3 (H3), and presented as fold changes relative to the control group (the level of the control group was set as 1). (**C**) and (**D**) FLSs were treated with or without FSTL1 for 12 hrs, and then ChIP assay targeting the p65 binding site on TNF-α (**C**) and IL-6 promoter (**D**) was performed. Data were shown as the percentage of input. The above results are presented as mean ± (standard deviation) SD of three independent experiments (each FLS cell line from an individual patient was used for each experiment). ^*^ *P* <0.05, ^**^ *P* <0.01, ^***^ *P* <0.001, compared to the vehicle-treated group, Student *t* test. ChIP, chromatin immunoprecipitation; FLS, fibroblast-like synoviocyte; FSTL1, follistatin-like protein 1; IL-6, interleukin-6; NF-κB, nuclear factor kappa B; PBS, phosphate-buffered saline; TNF-α, tumor necrosis factor alpha.

**Figure 3:**
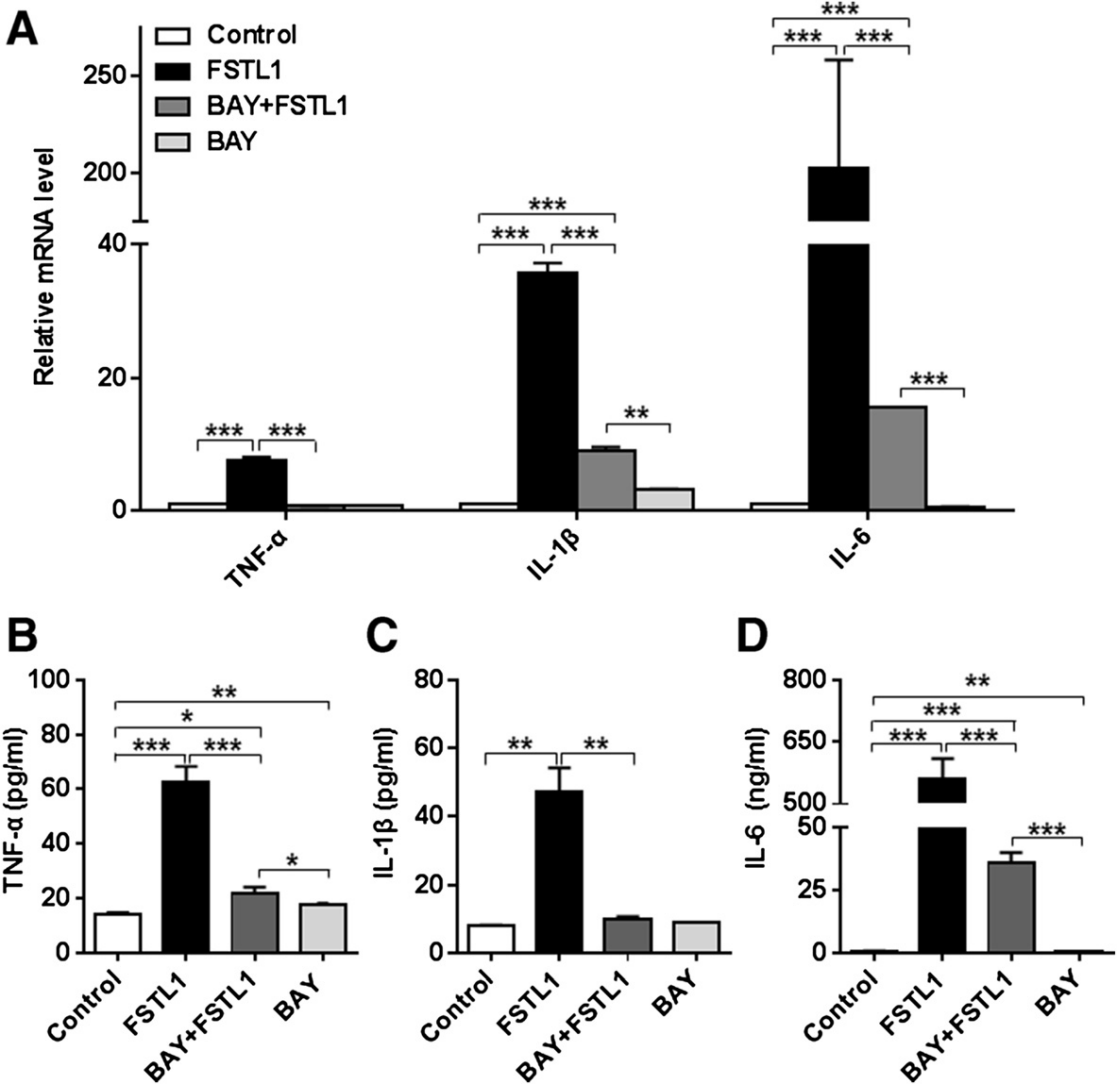
Inhibition of the NF-κB pathway eliminates the proinflammatory effects of FSTL1. (**A**) FLSs cultured in a 6-well plate were pretreated with BAY 11–7082 for 1 hour before addition of FSTL1, and then incubated with FSTL1 or vehicle (PBS solution) for 48 hrs. The relative levels of TNF-α, IL-1β and IL-6 mRNA were analyzed by real-time PCR as in Figure 1. (**B**-**D**) Cell supernatants were collected, and TNF-α, IL-1β and IL-6 concentrations were evaluated by ELISA. All results are presented as mean ± (standard deviation) SD of three independent experiments (each FLS cell line from an individual patient was used for each experiment). ^*^ *P* <0.05, ^**^ *P* <0.01, ^***^ *P* <0.001, relative to the control or between groups as indicated, Student *t* test. ELISA, enzyme-linked immunosorbent assay; FLS, fibroblast-like synoviocyte; FSTL1, follistatin-like protein 1; IL-1β, interleukin-1β; IL-6, interleukin-6; NF-κB, nuclear factor kappa B; PBS, phosphate-buffered saline; PCR, polymerase chain reaction; TNF-α, tumor necrosis factor alpha.

NF-κB signaling is a canonical pathway that participates in immunity and inflammation [28,29]. Thus we wanted to know whether FSTL1 exerts its inflammatory effects by activation of NF-κB signaling. By western blot analysis, we found that the level of phosphorylated p65 (p-p65) and phosphorylated IκBα (p-IκBα) in the total cell lysate were significantly increased following 5 μg/ml FSTL1 stimulation for 30 min (Figure 2A). In contrast, the expression of NF-κB subunit p65 and its intracellular inhibitor IκBα did not show significant changes (Figure 2A). Next, we separated the contents of the nucleus and carried out western blot analysis. A marked increase of p-p65, but not p50/105, was observed in the nucleus (Figure 2B). To further link NF-κB activation to downstream transcriptional activity, we performed ChIP assay. As compared to the control, FSTL1 promoted more p65 subunits to bind to the TNF-α and IL-6 promoter region, suggesting transcription activation of TNF-α and IL-6 (Figure 2C-D). And the enrichment folds were approximately 1.5 and 8 respectively, in line with the pattern of the mRNA increments. Next, we utilized BAY 11–7082 (5 μM), an effective inhibitor of IκBα phosphorylation, to specifically abrogate NF-κB DNA binding, and eventually downregulate the NF-κB-inducible cytokine levels. Compared to the control group, BAY 11–7082 significantly attenuated the FSTL1-triggered increase in the expression of inflammatory cytokines (Figure 3A-D). And the inhibitor itself did not affect the baseline level of these cytokines. Taken together, FSTL1 contributes to the proinflammatory effects by activating NF-κB signaling in OA FLS.

### FSTL1 accelerates the proliferation of FLS in a p53 and p21 dependent way

**Figure 4:**
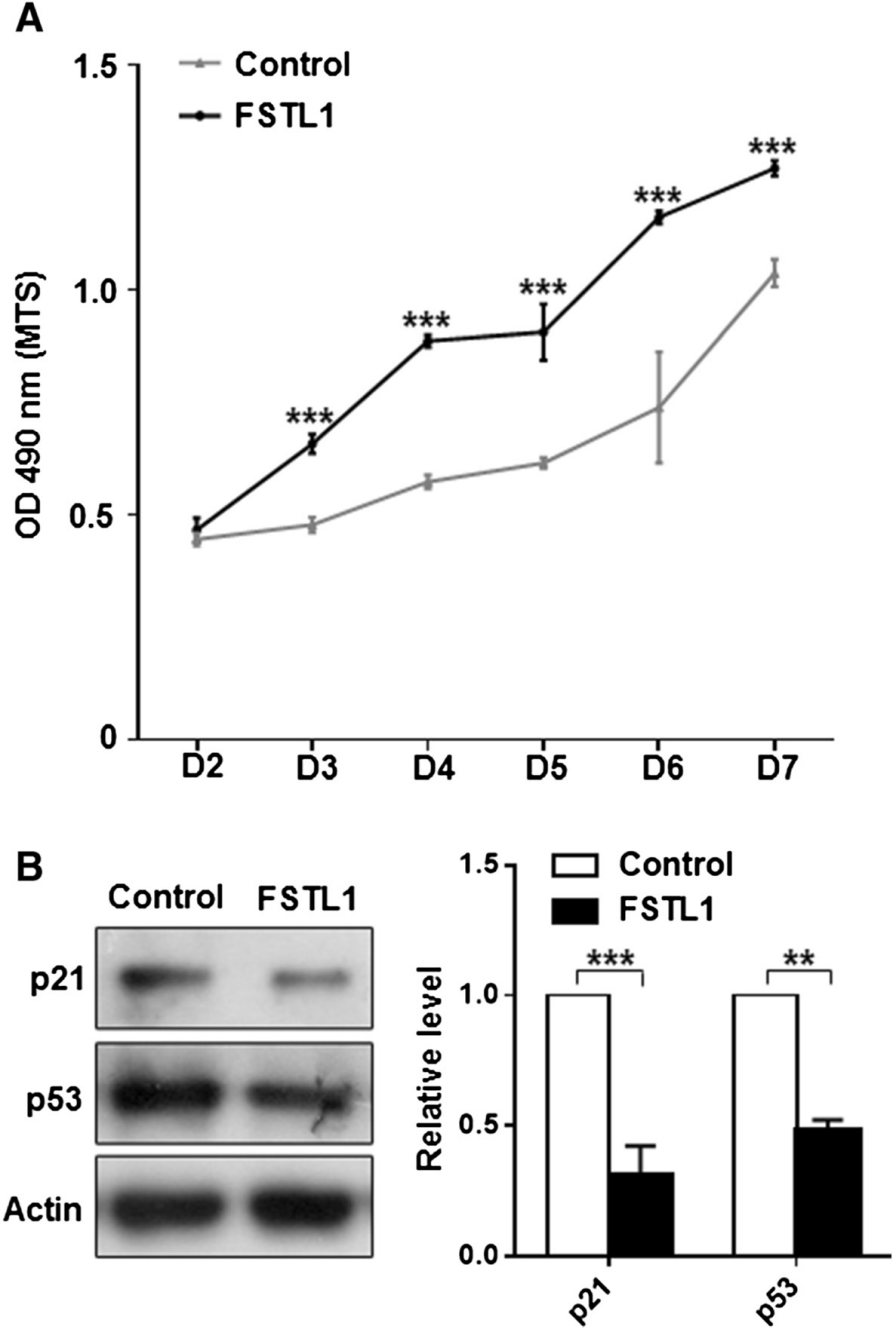
FSTL1 enhances proliferation of FLS in a p53- and p21-dependent way. (**A**) About 2,000 OA FLS cells per well were seeded into a 96-well plate in triplicate (the seeding day was referred to as Day 0, that is, D0). On the subsequent 6 days (referred as to D1 to D6), 5 μg/ml FSTL1 was daily applied to the medium. MTS test was performed daily on D2 to D7. (**B**) Cells were collected after D4 exposure to 5 μg/ml FSTL1 and western blots for p53 and p21 were conducted. Left panel, representative blots; Right panel, quantification data. Results in (**A**) and (**B**) are presented as mean ± (standard deviation) SD of three independent experiments (each FLS cell line from an individual patient was used for each experiment). ^*^ *P* <0.05, ^**^ *P* <0.01, compared to the vehicle-treated control, Student *t* test. FLS, fibroblast-like synoviocyte; FSTL1, follistatin-like protein 1; MTS, 3-(4,5-dimethylthiazol-2-yl)-5-(3-carboxymethoxyphenyl)-2-(4-sulfophenyl)-2H-tetrazolium, inner salt; OA, osteoarthritis.

Previous work suggests that activation of the NF-κB signaling pathway is associated with synovial fibroblast hyperplasia, and thus contributes to OA progression [30]. Thus, we wondered if FSTL1 promotes the proliferation of FLS. After FLSs were treated with FSTL1 for 6 consecutive days (FSTL1 was daily added to the medium), an increase of FLS number was significant in the subsequent 5 days (D3 to 7) as indicated in Figure 4A. To further examine if the FSTL1-induced FLS proliferation depends on the expression of two tumor suppressors: p53 and p21, we carried out western blot analysis on D4 and D6. As shown in Figure 4B, FSTL1 significantly inhibited the expression of p53 and p21 on the two time points (the data from D6 not shown). Collectively, these results suggest that FSTL1 enhances the proliferation of FLS in a p53- and p21-dependent way in OA FLS.

### The elevated level of SF FSTL1 and serum FSTL1 correlates with the increased level of SF IL-6 in OA patients

**Figure 5:**
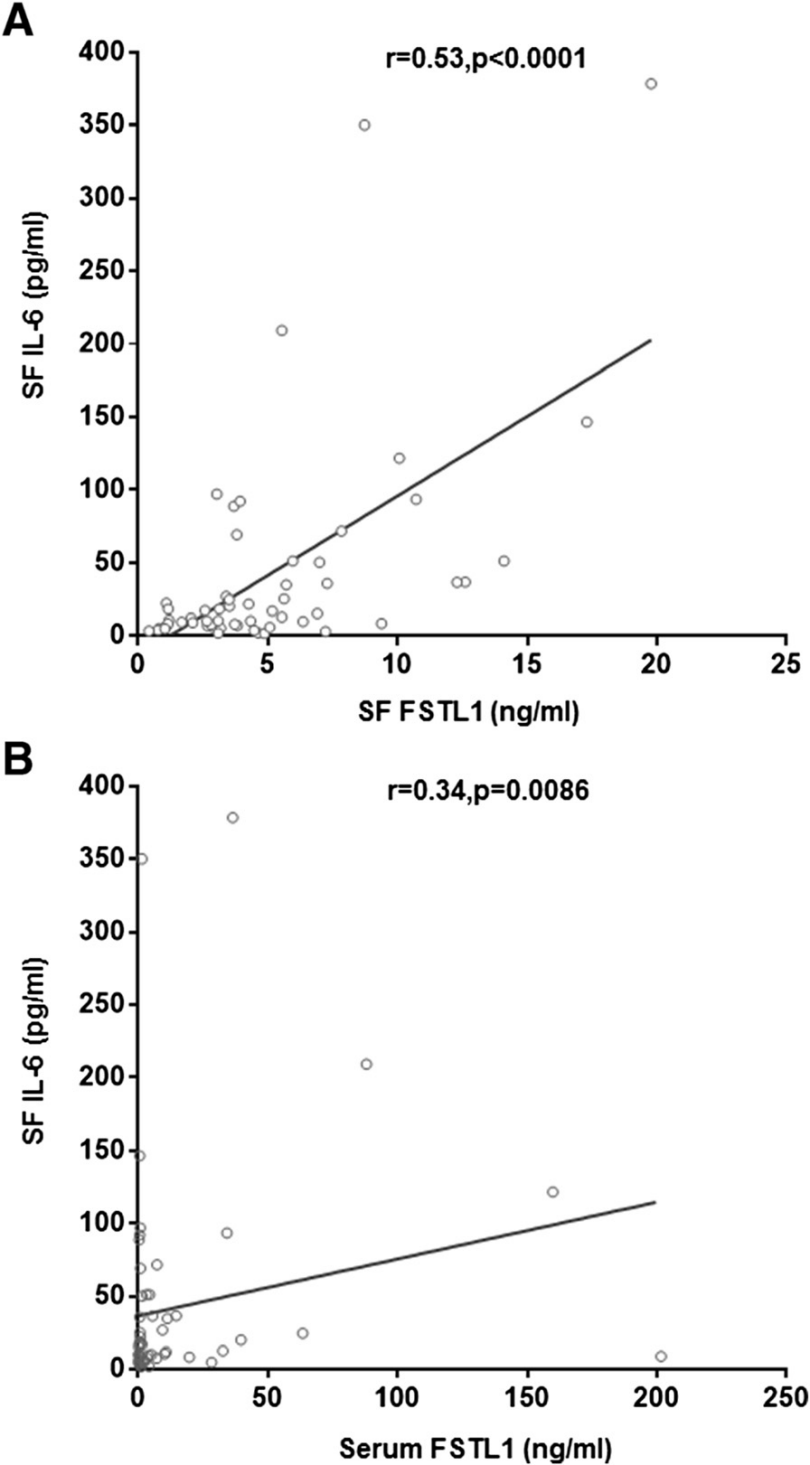
Correlation of SF and serum FSTL1 concentrations with SF IL-6 concentration in OA patients. (**A**) The association between concentrations of IL-6 and FSTL1 in SF of OA patients was evaluated. (**B**) The association between concentrations of SF IL-6 and serum FSTL1 in OA patients was evaluated. Fifty-eight paired samples (serum versus SF) were collected from 58 OA patients. Correlation coefficients (r) and *P* values from the Spearman rank-order test are shown. Each point represents an individual value. FSTL1, follistatin-like protein 1; IL-6, interleukin-6; OA, osteoarthritis; SF, synovial fluid.

As shown earlier, FSTL1 significantly improved expression and release of three inflammatory cytokines. In combination with our previous work that both serum and SF levels of FSTL1 are elevated in OA patients [24]. First, we tried to compare the concentrations of these cytokines in serum between OA patients and HC. By ELISA analysis, the three cytokines (TNF-α, IL-1β and IL-6) were undetectable in all HC serum samples. Also, we failed to detect the levels of TNF-α and IL-1β in OA patients. Intriguingly, we observed a marked increase of the SF IL-6 level (42.08 ± 73.38 pg/ml) in 58 OA patients. Next, we analyzed the association between FSTL1 (SF and serum) and SF IL-6 in 58 OA patients. As illustrated in Figure 5A-B, the level of SF IL-6 is significantly associated with SF FSTL1(r = 0.53, *P* <0.0001) and also correlated with serum FSTL1 (r = 0.34, *P* = 0.0086). These data suggest that FSTL1 is involved in osteoarthritis by elevating inflammatory cytokine IL-6.

## Discussion

As a secreted extracellular glycoprotein, FSTL1 is widely considered to participate in development and immune diseases pathogenesis. And some groups identified FSTL1 as a new proinflammatory mediator that contributes to rheumatoid arthritis by promoting the expression of TNFα, IL-1β, IL-6 and IL-8, as well as by enhancing the interferon-γ (IFN-γ) signaling pathway in a mouse model [22,31,32]. In this study, we observed two significant effects of FSTL1 in cultured OA FLS. One is that FSTL1 remarkably stimulates the expression and secretion of proinflammatory cytokines TNF-α, IL-1β, and especially IL-6 by activating the NF-κB signaling pathway. Interestingly, there was an association between elevated FSTL1 levels and significant increased IL-6 concentrations in SF of OA patients. The other one is that FSTL1 promotes the proliferation of FLS in a p53- and p2-dependent way. These findings further suggest that FSTL1 might be a potential target for the treatment of OA in humans.

TNF-α, IL-1β and IL-6 are thought to be the key proinflammatory cytokines related to the pathophysiology of OA [33]. A number of studies revealed that the function of TNF-α is involved in activation of the inflammatory cascades and IL-1β is a major player of cartilage erosion in OA [34-37]. IL-6 is also reported to exert a proinflammatory role in degenerative joint disease [37]. Importantly, the serum and SF levels of IL-6 are elevated in OA patients [38,39], consistent with our results. Moreover, IL-6 attributes to subchondral bone damage in OA by triggering osteoclast differentiation and bone resorption [40]. Our prior work showed that the level of increased serum FSTL1 may be identified as a potential serum marker related to the severity of joint damage of OA [23]. Taken together, FSTL1-induced IL-6 overexpression plays a key role in exaggerating OA progression. In this study, we further established the correlation between serum FSTL1 level and IL-6 concentrations in SF. FSTL1 exerts its proinflammatory role by changing the microenvironment of synoviocytes and directly stimulating secretion of inflammatory cytokines by FLS.

To further understand the role of FSTL1 in OA progression, we aimed to search for its receptor on FLS membrane. Murakami and his coworkers reported that a disco-interacting protein 2 homolog a (DIP2a) from *Drosophila* was a candidate receptor for FSTL1 in endothelial cells [39]. It exerted the anti-apoptotic and promigratory effects of FSTL1 and mediated FSTL1-induced activation of Akt [41,42]. But so far there are no reports of the existence of DIP2a in synovium or FLSs. In our primary FLS culture, only a few of DIP2a-positive cells were detected by immunohistochemistry staining. Furthermore, we knocked down of DIP2a with small interfering RNA (siRNA) (the effectiveness of knockdown was confirmed in HEK293T cells), but failed to observe statistically significant alterations in FSTL1-induced inflammatory cytokines. Therefore, it seems that the effects of FSTL1 may not be mediated by DIP2a in OA FLS. There is evidence that FSTL1 can bind to CD14 and elicits an innate immune response via Toll-like receptor 4 (TLR4) [43]. In HEK293T, FSTL1 activates NF-κB and elevates the level of IL-6 and IL-8 [43]. Thus it is likely that in OA FLS, FSTL1 promotes inflammation via the CD14-TLR4 pathway rather than DIP2a. In addition, we cannot exclude that the effects of FSTL1 is achieved via the TGF superfamily, since FSTL1 interacts with the TGF-β superfamily, including activin, TGF-β, bone morphogenetic protein (BMP)-2⁄4, and their receptors [42,44].

Our results show that FSTL1 activates the NF-κB pathway upon binding to its unidentified receptor in OA FLS. We provide evidence that the active transcriptional element p65 is recruited to the promoter region of TNF-α and IL-6, and as a result directly enhances the expression level of these cytokines. Since TNF-α and IL-1β also can be induced by activation of p38 and c-Jun N-terminal kinase (JNK) and their downstream transcriptional factor AP1 [45], it may be an explanation of the not fully abolished level of the cytokines after inhibition of NF-κB signaling by BAY 11–7082. Furthermore, it was reported that FSTL1 contributes to arthritis as a proinflammatory mediator by enhancing the IFN-γ signaling pathway in a collagen-induced arthritis (CIA) mouse model [32]. Overall, FSTL1 can activate multi-proinflammatory pathways to participate in OA pathophysiology.

In this study, we also observed that FSTL1 enhances FLSs proliferation, suggesting that FSTL1 acts as a synoviopathy-inducing factor to be involved in OA pathogenesis by the increasing expansiveness of FLS. It is thought that a pannus formed by abnormal proliferative FLS is a hallmark of rheumatoid arthritis and OA, finally damaging articular bone and cartilage [46]. Since p53, a tumor-suppressor protein, plays a pivotal role in cell cycle regulation and programmed cell death, we intended to determine whether the overgrowth is due to the inhibition of its activity in the presence of FSTL1. Our western blot analysis supported the idea. Supportably, p53 serves as an intracellular anti-inflammatory function [47]. The absence of p53 is involved in both decreased apoptosis of FLS and increased synovial IL-6 production in a CIA mouse model [48]. p21, a cyclin-dependent kinase inhibitor, is induced by p53, and participates in the pathogenesis of OA by blocking of overexpressed IL-6 and matrix metalloproteinases (MMPs) synthesis [49]. Consistently, we also observed that the application of FSTL1 markedly downregulated the expression of p21, implicating the involvement of p21 in the proliferative effects of FSTL1.Therefore, we propose that FSTL1 exacerbates OA by facilitating expansiveness of FLS as a synoviopathy-inducing factor besides increasing secretion of inflammatory cytokines. Like many inflammatory factors (such as interleukin-17 (IL-17)) [50], FSTL1 has ‘dual power’ to exacerbate arthritis.

## Conclusions

FSTL1 significantly promotes the expression of inflammatory cytokines (TNF-α, IL-1β and IL-6) in cultured FLS from OA patients, which is achieved by activating the NF-κB pathway. Moreover, FSTL1 improves FLS proliferation in a p53- and p21-dependent pathway. Finally, the elevated concentration of SF IL-6 is correlated with serum and SF FSTL1 levels in OA patients. These findings suggest that FSTL1 is involved in the progression of synovial inflammation and hyperplasia in OA, and presumably is a potential target for OA therapy.

## Abbreviations

BMP: bone morphogenetic protein
ChIP: chromatin immunoprecipitation
CIA: collagen-induced arthritis
DIP2a: disco-interacting protein 2 homolog a
DMEM: Dulbecco’s modified Eagle’s medium
DMSO: dimethyl sulphoxide
ECM: extracellular matrix
ELISA: enzyme-linked immunosorbent assay
FBS: fetal bovine serum
FLS: fibroblast-like synoviocyte
FSTL1: follistatin-like protein 1
GAPDH: glyceraldehyde 3-phosphate dehydrogenase
HC: healthy control
IgG: immunoglobulin G
IκB: inhibitor of kappa B
IKK: IκB kinases
IFN-γ: interferon-γ
IL-1β: interleukin-1β
IL-6: interleukin-6
IL-8: interleukin-8
IL-17: interleukin-17
JNK: c-Jun N-terminal kinase
KL: Kellgren-Lawrence
MMP: matrix metalloproteinase
MTS: 3-(4,5-dimethylthiazol-2-yl)-5-(3-carboxymethoxyphenyl)-2-(4-sulfophenyl)-2H-tetrazolium, inner salt
NF-κB: nuclear factor kappa B
OA: osteoarthritis
PBS: phosphate-buffered saline
PCR: polymerase chain reaction
PES: phenazine ethosulfate
PVDF: polyvinylidene fluoride
SF: synovial fluid
SDS: sodium dodecyl sulfate
siRNA: small interfering RNA
TGF-β1: transforming factor β1
TLR4: Toll-like receptor 4
TNF-α: tumor necrosis factor alpha
